# Optimization of Heat Treatment for 38Si7 Spring Steel with Excellent Mechanical Properties and Controlled Decarburization

**DOI:** 10.3390/ma15113763

**Published:** 2022-05-24

**Authors:** Xian-Wen Wang, Qing-Feng Hu, Chao-Lei Zhang, Lie Chen, Chang-Yong Zhu, Bo Tao, Bo Jiang, Ya-Zheng Liu

**Affiliations:** 1 School of Materials Science and Engineering, University of Science and Technology Beijing, Beijing 100083, China; s20200362@xs.ustb.edu.cn (X.-W.W.); huqingfeng_ustb@163.com (Q.-F.H.); jiangbo@ustb.edu.cn (B.J.); lyzh_ustb@163.com (Y.-Z.L.); 2 Jianlong Beiman Special Steel Co., Ltd., Qiqihar 161041, China; ccmo_tu@163.com; 3 China Railway Longchang Equipment Co., Ltd., Neijiang 642150, China; ke77.op@gmail.com (C.-Y.Z.); ui669841@163.com (B.T.)

**Keywords:** orthogonal experiment, heat treatment, microstructure, mechanical properties, fractography, surface decarburization

## Abstract

Optimizing the heat treatment procedure with 13 mm diameter 38Si7 spring steel is critical for developing high-performance, low-cost, large spring steel for railway clips. The effects of quenching temperature, holding time, tempering temperature, and tempering time on the microstructure and mechanical properties were investigated using an orthogonal experiment, designed with four factors and three levels. The best heat treatment settings were explored, as well as the variation laws of mechanical properties, decarburization behavior, and fracture morphology. The results demonstrated that quenching temperature and tempering temperature had the most impact on plasticity and tempering temperature, while time had the most effect on strength. The optimized heat treatment schemes made the elongation increase by up to 106% and the reduction in area increase by up to 67%, compared with the standard BS EN 10089-2002, and there were mixed fractures caused by ductility and brittleness. The fracture tests showed a good performance of 20.2 GPa·%, and the heat treatment processes’ minimum decarburization depth of 93.4 μm was determined. The optimized process would obtain stronger plastic deposition and better decarburization performance. The microstructure was simply lightly tempered martensite, and the matrix still retained the acicular martensite. The optimal heat treatment process is quenching at 900 °C for 30 min (water cooling), followed by tempering at 430 °C for 60 min (air cooling). The research led to a solution for increasing the overall mechanical characteristics and decreasing the surface decarburization of 38Si7 spring steel with a diameter of 13 mm, and it set the foundation for increasing the mass production of railway clips of this size.

## 1. Introduction

Rail transportation has always been essential in transportation in many countries and regions, and it has contributed to boosting the degree of national economic growth [1]. According to statistics, the United States’ railway mileage has ranked top in the world recently, with a total length of over 220,000 km. China ranked second, with an overall length of 150,000 km, and China’s high-speed rail mileage ranked first in the world, with a total length of 20,000 km, accounting for around 65% of the world’s high-speed rail mileage. Other countries’ railway lines are also responsible for a significant proportion. The spring clips for high-speed railway tracks are important in assuring railway transportation’s safety, efficiency, and comfort [2]. They are responsible for providing a secure connection [3] between the rails, preserving track integrity, limiting longitudinal and transverse rail movement relative to the sleeper, reducing the vibration of the system [4,5], and maintaining normal gauges [6]. This requires a more stringent standard for the clips’ strength and fatigue resistance. In addition, a set of spring fasteners must be installed every 60~80 cm along the railway track, which indicates that at least 1250 sets of spring bars are required per kilometer of railway. Railway fasteners are in high demand due to the vast application market. Therefore, developing high-performance, low-cost spring steel for railway spring clips is important.

Currently, the most common spring steels are 60Si2Mn [7,8,9,10], 60Si2MnWE [11], 60Si2CrA [12], 51CrV4 [13,14,15], 38Si7 [8,10], etc. Their chemical constituents are shown in Table 1. Since the medium-carbon spring steel 38Si7 contains less carbon than high-carbon spring steels, it has reduced decarburization sensitivity and improved fatigue resistance. Moreover, 38Si7 spring steel has no microalloying components. It is appropriate for today’s worldwide demands for resource conservation and carbon neutrality. The issue is that BS EN 10089-2002 only mentions 38Si7, and the standard only defines the procedures for products with a diameter of (Φ9) 9 mm. To expand the applicability of spring steel—especially for the vast Chinese market—the performance requirements of the Φ13 mm spring clips specification should be met. This might also shorten the length of the sleeper and greatly reduce the cost of railway construction. As is known to all, increasing the size of steel leads to a large decline in performance if the same process is adopted [16]. This study focuses on the research of Φ13 mm railway elastic strips to improve the comprehensive performance accordingly.

Considerable efforts have gone into developing high-performance spring steel over several decades. Heat treatment processes [17,18,19,20,21,22] and microalloying [18,23] are the most effective for intensifying the strength limit. There are, however, only a few systematic studies on the heat treatment of 38Si7 steel with the Φ13 mm specification. The difficulty arises from the loss of strength and plasticity [24] during heat treatment, as well as the significant decarburization of the surface [25,26]. The procedure must be optimized without raising the cost to obtain better overall mechanical properties and control the decarburization of 38Si7. Related research has shown that quenching and tempering factors determine the final mechanical properties and fatigue resistance by changing the microstructure [27,28]. However, the majority of current studies on 38Si7 are on fatigue fracture [28,29] and the stress state simulation of finished products [30,31]. It is critical to investigate the relationships between the processes and properties of large 38Si7spring steel.

Thus, this study aims to optimize the heat treatment procedure of 38Si7 steel with Φ13 mm, and the relationship between the quenching factor and tempering factor with respect to the mechanical properties, microstructure, fracture morphology, and decarburization behavior is established to increase the service performance of 38Si7 spring steel in railway fasteners.

## 2. Experimental Procedures

**Figure 1:**
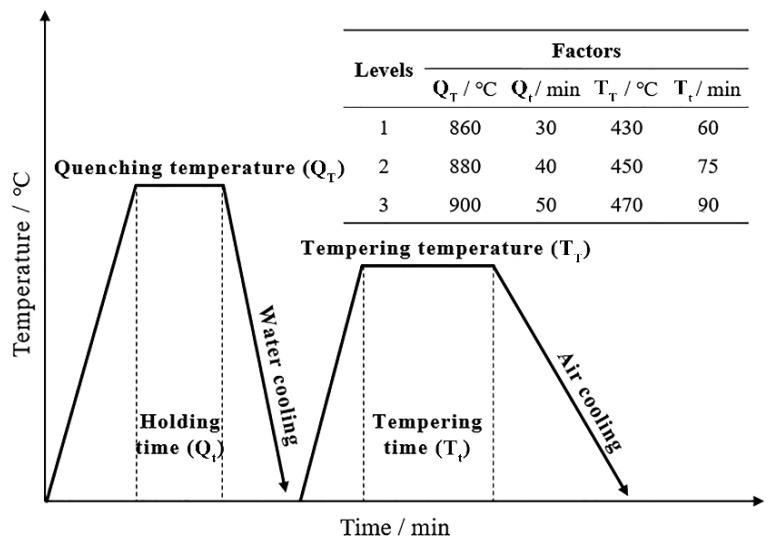
The quenching and tempering heat treatment process of Φ13 mm 38Si7 spring steel, and the four-factor three-level method.

The experimental steel was 38Si7 spring steel, which was obtained in a commercial hot-rolled wire 13 mm in diameter from a high-speed wire mill. The A_c1_ and A_c3_ of the experimental steel were 765 °C and 797 °C, respectively. The wire rod was processed into specimens of Φ13 mm × 60 mm and then cut off at a half arc at 4 mm from the center. Machining marks on the surface of the specimens were sanded away before the subsequent heat treatments. The wire rod was cut into Φ13 mm × 10 mm specimens, and then a half arc was cut off 4 mm from the center. Before the heat treatment, machining marks on the surface of the specimens were polished away. The heat treatment was designed by using the orthogonal experiment method with L9 (3^4^), and four factors were analyzed: quenching temperature (Q_T_), holding time (Q_t_), tempering temperature (T_T_), and tempering time (T_t_), as shown in Figure 1. Water cooling after quenching and air cooling after tempering were carried out for all samples. Three levels were created for each factor. The heat treatment was carried out in an SGM·M30/12 box furnace (SGM, Luoyang, China).

**Figure 2:**
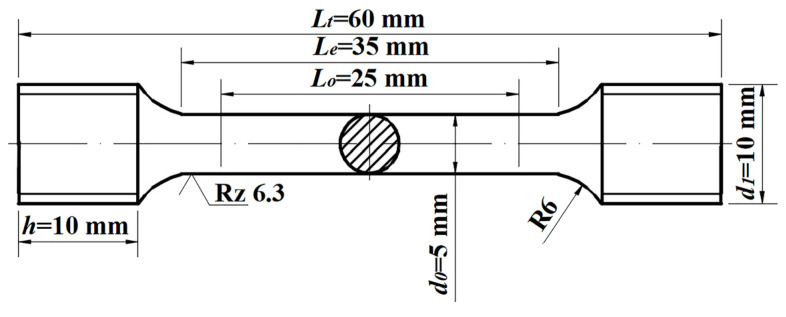
The shape of the mechanical properties test specimen.

The mechanical property test samples after tempering are shown in Figure 2. They conform to the standard GB/T 228.1-2010 (ICS 77.040.10). The surface of the mechanical property test samples was turned 2 mm before the test to remove the influence of the decarburization layer on the performance. The experiment used the CMT-4105 tensile tester (CMT-4105, Shanghai, China) to determine mechanical properties at room temperature, and the three groups of data were averaged to reduce the error. The specimens were polished and etched in 3% nitric acid solution (3 mL of nitric acid dissolved in 97 mL of ethanol). A scanning electron microscope (Zeiss Gemini SEM 500 and FEI Quanta 250 (JEOL, Tokyo, Japan)) was used to analyze the microstructure. The decarburized depth was measured using the micrographic method in accordance with China GB/T 224-2019.

## 3. Results and Discussions

### 3.1. Orthogonal Experiments

Table 2 shows the experimental results of nine different heat treatments according to the orthogonal experimental design. The values in the table are the average values obtained from three tests to reduce the error. The mechanical properties include ultimate tensile strength (UTS), yield strength (YS), elongation (A_5_), and reduction in area (Z). The requirements of the standard BS EN 10089-2002 are also presented in Table 2. The ultimate tensile strength of processes No.3, No.6, and No.9 is less than 1300 MPa. The yield strength of the No.3 process is less than 1150 MPa. It is worth mentioning that the A_5_ of some results increased by up to 106%, and the Z increased by up to 67%, compared with the standard BS EN 10089-2002.

**Figure 3:**
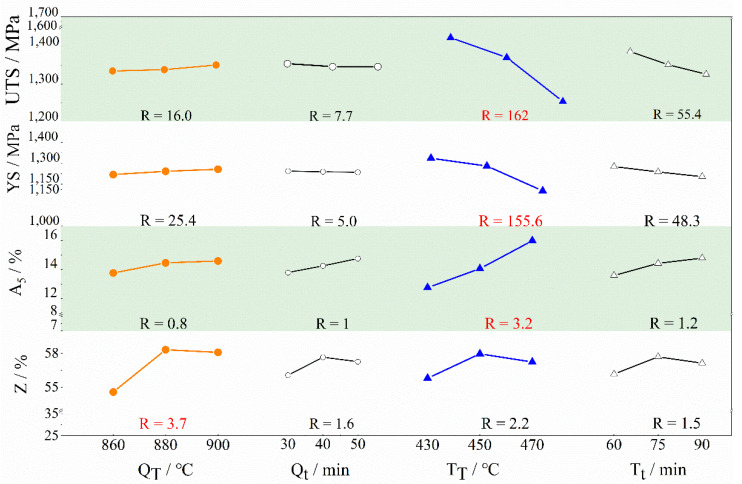
Factors and index trend chart of range analysis: quenching temperature (Q_T_), holding time (Q_t_), tempering temperature (T_T_), tempering time (T_t_).

The influence of four factors and indices evaluated using the range analysis approach is displayed in Figure 3. The value of each index of the ordinate in the figure was obtained according to the range evaluation method in the orthogonal experiment, and the values represent the average values of all measured values in Table 2 under these conditions. A larger range value suggests that the factor has a stronger influence on mechanical properties. The following was the order in which the four factors affected the properties: UTS (T_T_ > T_t_ > Q_T_ > Q_t_), A_5_ (T_T_ > T_t_ > Q_t_ > Q_T_), Z (Q_T_ > T_T_ > Q_t_ > T_t_).

According to the above findings, tempering temperature had the most impact on ultimate tensile strength, yield strength, and elongation, while quenching temperature had the greatest impact on the reduction in area. The elongation of all orthogonal tests was better than the standard 3.1~8.5%, and the reduction in the area was greater than the standard 16.6~23.5%. The ultimate tensile strength and yield strength of the tempered samples at 430 °C and 450 °C met the specification. Furthermore, it could be predicted that the ultimate tensile strength and yield strength would decrease with increasing holding time and tempering temperature, but would rise with increasing quenching temperature. Investigating the mechanisms of quenching and tempering factors from the standpoint of microstructural transformation is critical to explaining this prediction.

### 3.2. Effects of Quenching on Microstructure and Mechanical Properties

#### 3.2.1. Size Evolution for Prior Austenite

**Figure 4:**
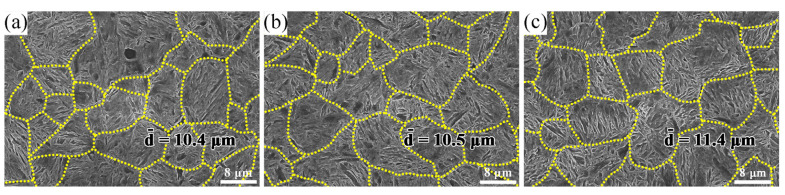
The prior austenite grain size at different quenching temperatures (**a**) 860 °C; (**b**) 880 °C; (**c**) 900 °C.

The micrographs of specimens after various heat treatments are shown in Figure 4, showing that they might affect their mechanical properties. Dotted lines were used to increase the visibility of the prior austenite grain boundary, and then at least 2000 pre-austenite grains in the other fields of view were measured and averaged. The average grain sizes of No.1, No.6, and No.8 were 10.4 μm, 10.5 μm, and 11.4 μm, respectively. The grain growth rate of austenite conformed to the Arrhenius model [32], as shown in Equation (1):

(1) $${D=A\cdot t}^{n}\cdot \exp(-\frac{Q}{R\cdot T})$$

where *D* is the austenite grain growth size, *A* represents the material constants, *t* is the holding time, *n* is the grain growth index, *Q* is the activation energy for grain growth, *R* is the gas constant, and *T* is the heating temperature.

The *T* increased as the quenching temperature rose, as did the austenite size *D*. Thus, the prior austenite grains continued to coarsen as the quenching temperature increased, which was consistent with the statistical data. The prior austenite grains exhibited minimal modification when the quenching temperature was less than 880 °C (Figure 4a,b), and the austenite grains developed when the quenching temperature approached 880 °C. When the quenching temperature exceeded 880 °C, the second-phase particles began to dissolve and the nailing effect weakened. Then, the austenite grains expanded. The prior austenite grains increased rapidly when the quenching temperature reached 900 °C. After quenching at 900 °C for 50 min, they were absorbed by large grains (Figure 4c).

The mechanical properties (Table 2) demonstrated that as the quenching temperature increased, so did the strength. On the one hand, the saturation of martensite could be improved with a higher quenching temperature. This could dissolve more carbides and alloying elements into the matrix, and the austenitizing process of experimental steel was accelerated. On the other hand, the prior austenite grains were divided into numerous martensite packets with the same habits, but different orientations. The packets then were separated into several blocks with similar orientations. The subgrain structures in the blocks were called laths, where the laddings and blocks belonged to a large-angle grain boundary and the lath to a small-angle grain boundary. The low-angle grain boundary could effectively hinder the dislocation motion, resulting in strengthening and reducing the amount of stress concentration. Therefore, the adjustment ability of the low-angle grain boundary to strength and plasticity was improved [33,34].

#### 3.2.2. Surface Decarburization Behavior

**Figure 5:**
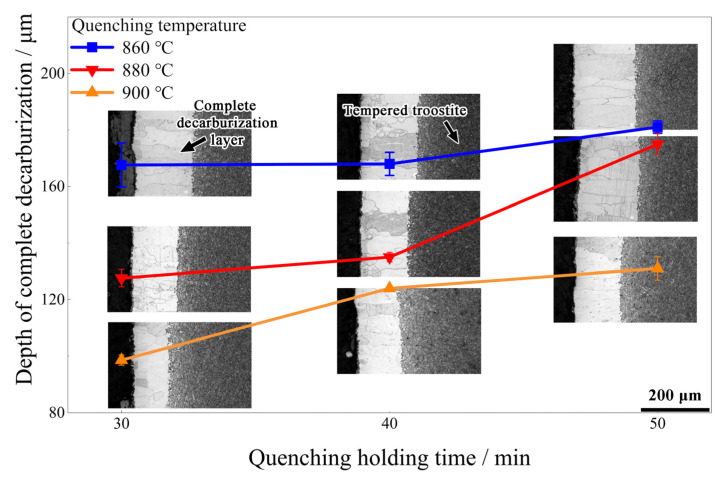
The depth of complete decarburization (DCD) and corresponding decarburization microstructure after heating treatment.

After heat treatment, there were obvious decarburization layers on the surface (Figure 5). The tempering temperature did not affect the depth of the complete decarbonization. The depth of decarburization is also shown in Figure 5. The decarburization diminished as the quenching temperature rose from 860 °C to 900 °C under the same holding time. When the quenching temperature remained constant, the depth of complete decarburization grew as the quenching holding time increased. The sensitive range of complete decarburization of 38Si7 was 725~875 °C [35]. When quenching at 900 °C, it exceeded the sensitive interval of complete decarburization. The depth of complete decarburization reached the minimum (93.4 μm) after 30 min of holding at this temperature. This was significantly less than the maximum depth of 195 μm specified in BS EN 10089-2002. The reduction of the decarburized layer could positively affect the material’s fatigue resistance and service performance [36,37,38,39].

### 3.3. Effects of Tempering Factors on Mechanical Properties and Fracture

#### 3.3.1. Mechanisms of Strength Change Caused by Tempering

**Figure 6:**
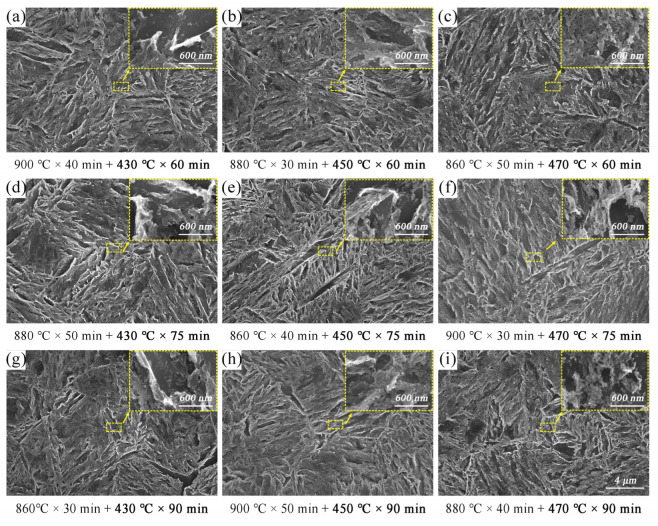
Microstructures corresponding to different tempering conditions: (**a**) No.7; (**b**) No.5; (**c**) No.3; (**d**) No.4; (**e**) No.2; (**f**) No.9; (**g**) No.1; (**h**) No.8; (**i**) No.6.

The microstructure of the samples after tempering is shown in Figure 6. The findings revealed that as tempering temperature and time increased, martensite decomposed into a large number of cementite sheets, promoting coarsening of the initial carbides. The microstructure was simply lightly tempered martensite, while the matrix was acicular martensite. When the tempering temperature increased from 430 °C to 470 °C, the continuous thin-film carbide at the lath interface or phase interface gradually decreased through decomposition and element enrichment size, showing a tendency toward spheroidization. The morphology of carbide did not change significantly when the tempering time increased from 60 min to 90 min at the same tempering temperature.

Tempering features (Figure 3 and Table 2) show that the strength of specimens decreased with increasing tempering temperature and time. The substructures of the dislocation provided the location for the nucleation of the carbide during tempering. Carbides would decompose and precipitate because of tempering. As a result, the sample exhibited a strong precipitation-intensifying effect [7,40], which improved the ultimate tensile strength. The relationship between dislocation density (*ρ*) and carbon concentration (*C*) in martensite could be expressed by the M–H equation [41] (Equation (2)). The amount of carbon element precipitation increased with tempering temperature. Due to this, the carbon concentration of the martensite decreased, as did its dislocation density. Thus, the UTS and YS reduced. The results of orthogonal experiments (Section 3.1.) also confirmed this.

(2) $${\rho \times 10}^{–15}=0.7+3.5\cdot C$$

#### 3.3.2. Fracture Characteristics

**Figure 7:**
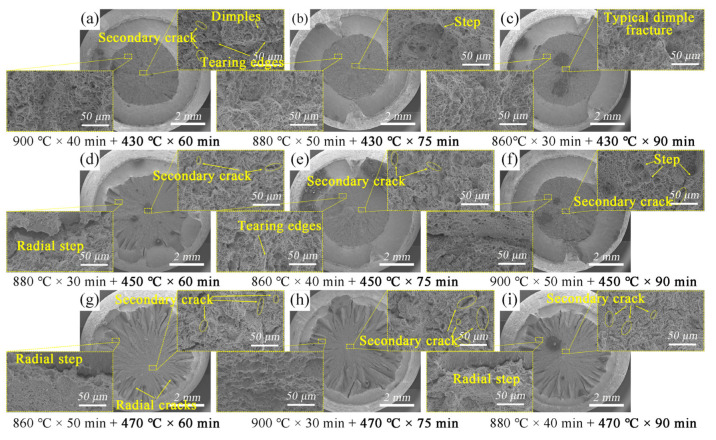
The fracture surface of tensile specimens corresponding to different tempering conditions: (**a**) No.7; (**b**) No.4; (**c**) No.1; (**d**) No.5; (**e**) No.2; (**f**) No.8; (**g**) No.3; (**h**) No.9; (**i**) No.6.

The fracture morphology under different heat treatment schemes is shown in Figure 7. When tempering at 430 °C, 450 °C, and 470 °C for 60 min, the proportion of the shear lip in the whole fracture surface was 58.5%, 41.2%, and 19.2%, respectively. The source of the crack was in the fiber zone of the fracture. The dimples in the fiber zone gradually decreased in size but became deeper with the increase in tempering temperature (Figure 7a,b,e). The fracture of the tensile samples changed from a mixed ductile–brittle fracture to a complete ductile fracture. The rapid decrease in internal stress in the grain boundary as the tempering temperature increased—especially the decomposition of martensite laths or plates and the reduction in dislocation—caused this change.

The fracture characteristics could better reflect the plasticity and performance change mechanisms of materials [42,43,44,45]. When the tempering temperature rose to 470 °C, huge radiation steps and deformed radial cracks were generated during the tensile tests (Figure 7g–i). The fiber region appeared a typical dimple fracture. The larger dimples were formed around inclusions, and smaller dimples were aggregated near carbides. In addition, there were dense tearing edges in the radiation zone of tiny size, and some of them were accompanied by tearing dimples. The sample tempered at 470 °C had the highest plasticity.

### 3.4. Selection of the Optimal Heat Treatment Process

**Figure 8:**
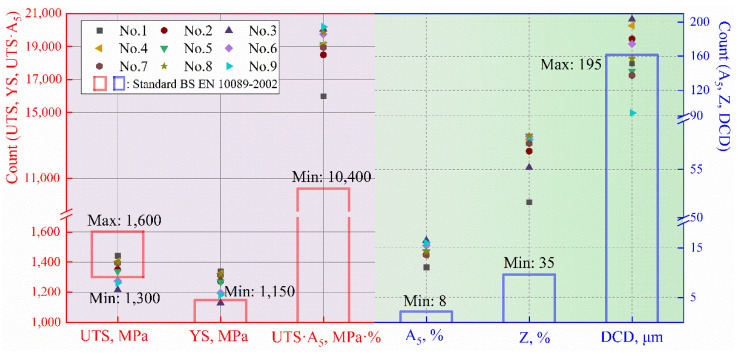
Comparison results of nine groups of experiments and the standard BS EN 10089-2002.

Based on the orthogonal experiment analysis (Figure 3), the heat treatment processes with the highest ultimate tensile strength and yield strength were 900 °C × 30 min and 430 °C × 60 min. The highest elongation was 900 °C × 50 min and 470 °C × 75 min.The highest reduction in area was 880 °C × 40 min and 450 °C × 75 min. The comparison between the experimental results and the standard is shown in Figure 8.

**Figure 9:**
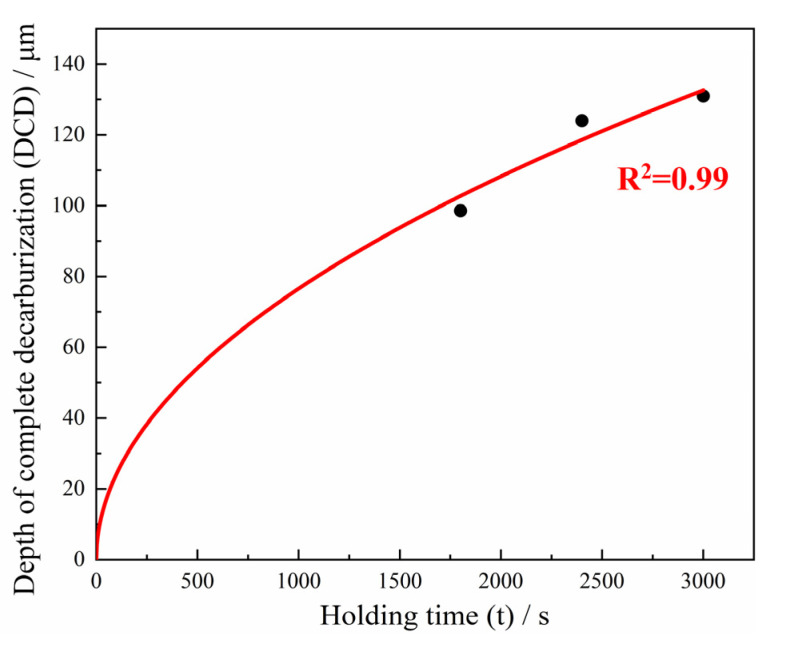
The variation law of complete decarburization depth at 900 °C.

Since the minimum depth of the decarburization layer is obtained at 900 °C, the key to controlling the decarburization is to reveal its evolution law. According to Figure 5, and combined with the literature [46], it can be known that when the heating temperature is constant, a mathematical model between actual decarburized depth and heating time can be established according to Fick’s first law, as given in Equation (3). In consequence, the decarburized depth follows a parabolic law with the increase in the heating time. According to the fitting results of the formula, as shown in Figure 9, the evolution law with the holding time at 900 °C is shown in Equation (4). This has guiding significance for controlling the decarburization layer of Φ13 mm 38Si7 spring steel.

(3) $$h_{\mathrm{DCD}}=k\cdot \sqrt{t}$$

where the *h*_DCD_ (μm) is the depth of complete decarburization, and the k is the constant coefficient, which can be obtained by fitting with the least squares method. The *t* (s) is the quenching holding time.

When quenching at 900 °C for 30 min (water cooling) and tempering at 470 °C (air cooling) for 75 min (No.9 process), the performance of facture work was as high as 20,192 MPa%. From the perspective of the product of tensile strength and elongation, the experimental results obtained an optimal result 9792 MPa% higher than that of the standard. In addition to excellent plasticity, it is important to consider the yield strength and the depth of complete decarburization [13,14,47]. Consequently, quenching at 900 °C for 30 min and tempering at 430 °C for 60 min is the best heat treatment process for Φ13 mm spring steel 38Si7.

(4) $$h_{\mathrm{DCD}}=2.42\cdot \sqrt{t}.$$

However, it is regrettable that this work fails to test the best scheme selected. Nevertheless, it is beneficial to elaborate on the correlation between the modifications in the microstructure, mechanical properties, decarburization behavior, and fracture characteristics after adjusting the quenching and tempering factors, and the conditions used in the laboratory are more precise, which may deviate from the actual production conditions, and these errors are expected to be solved in future research. This work could supply data to help improve the service performance of large Φ13 mm 38Si7 spring steel.

## 4. Conclusions

(1). This work proves the influence of quenching and tempering factors on the mechanical properties of Φ13 mm 38Si7 spring steel. The ultimate tensile strength and yield strength were T_T_ > T_t_ > Q_T_ > Q_t_; elongation was T_T_ > T_t_ > Q_t_ > Q_T_; and the reduction in area was Q_T_ > T_T_ > Q_t_ > T_t_. This provides evidence for future, in-depth research,, as well as a research foundation for accurately regulating the steel’s overall performance.
(2). When the quenching temperature exceeded 900 °C, the original austenite grains increased rapidly. The ultimate tensile strength and yield strength increased with quenching temperature. When the quenching temperature was between 860 °C and 900 °C, the depth of complete decarburization reduced monotonically as the temperature increased and the holding time decreased. The minimum depth of complete decarburization was 93.4 μm after quenching at 900 °C for 30 min.
(3). The ultimate tensile strength and yield strength decreased as the tempering temperature increased between 430 °C and 470 °C, and decreased as the tempering time increased. The microstructure was simply lightly tempered martensite, and the matrix was acicular martensite. The heating process would make the carbide spheroidization obvious, and the fracture characteristics also changed from a mixed ductile–brittle fracture to a ductile fracture. The study of the fracture showed a good performance of 20.2 GPa% GPa·% after tempering at 470 °C for 75 min.
(4). The optimal heat treatment scheme was determined as follows: quenching at 900 °C for 30 min and tempering at 430 °C for 60 min. This scheme aims to improve the comprehensive performance of Φ13 mm 38Si7 spring steel and promote the applicability of 38Si7 in railways.

## Figures and Tables

**Table 1:** The chemical composition of some spring steels (wt%).

	C	Mn	Si	Ni	Cr	W	Mo	V	Fe
60Si2MnWE	0.65	0.88	1.80	0.08	0.24	0.96	0.04	/	Bal.
60Si2MnA	0.60	0.75	1.69	/	0.14	/	/	/	Bal.
62Si2CrA	0.61	0.70	1.75	0.01	0.83	/	/	/	Bal.
51CrV4	0.56	0.69	0.30	/	1.07	/	/	0.14	Bal.
38Si7 (this work)	0.39	0.68	1.73	0.02	0.21	/	/	/	Bal.

**Table 2:** The orthogonal tables L9 (3^4^) and mechanical properties of 38Si7spring steel.

Process No.	Q_T_/°C	Q_t_/min	T_T_/°C	T_t_/min	UTS/MPa	YS/MPa	A_5_/%	Z/%
1	860	30	430	90	1440	1338	11.1	51.6
2	860	40	450	75	1350	1272	13.7	56.9
3	860	50	470	60	1215	1129	16.5	55.2
4	880	50	430	75	1404	1323	13.6	58.1
5	880	30	450	60	1338	1268	14.3	58.5
6	880	40	470	90	1274	1195	15.5	58.4
7	900	40	430	60	1393	1312	13.6	57.7
8	900	50	450	90	1398	1321	14.2	58.5
9	900	30	470	75	1262	1182	16.0	58.1
Standard BS EN 10089-2002					1300~1600	≥1150	≥8	≥35
